# Diagnostic and prognostic value of the Creatinine/Cystatin C ratio for low muscle mass evaluation among US adults

**DOI:** 10.3389/fnut.2022.897774

**Published:** 2022-08-09

**Authors:** Shanshan Shi, Yizhou Jiang, Weihua Chen, Kaihong Chen, Ying Liao, Kun Huang

**Affiliations:** ^1^Department of Cardiology, Longyan First Affiliated Hospital of Fujian Medical University, Longyan, China; ^2^Department of Internal Medicine, The Third Clinical Medical College, Fujian Medical University, Longyan, China; ^3^State Key Laboratory of Cardiovascular Disease, Fuwai Hospital, National Center for Cardiovascular Diseases, Chinese Academy of Medical Sciences and Peking Union Medical College, Beijing, China; ^4^Department of Industrial Engineering, Center of Statistical Science, Tsinghua University, Beijing, China

**Keywords:** sarcopenia, Creatinine/Cystatin C ratio, creatinine, cystatin C, low muscle mass, mortality

## Abstract

**Background:**

Identifying patients with low muscle mass is crucial for the diagnosis of sarcopenia. Although the Creatinine/Cystatin C (Cr/CysC) is recommended as a simplified indicator to identify patients with low muscle mass, its ability to assess muscle mass and predict a poor prognosis has not been validated. We aimed to determine the diagnosis value of Cr/CysC for low muscle mass and examine the association of Cr/CysC with mortality.

**Methods:**

In this cohort study we analyzed data from the National Health and Nutrition Examination Survey from 1999 to 2002. Follow-up was conducted up to December, 2015. Appendicular skeletal mass was calculated based on dual-energy X-ray absorptiometry (DXA) scans. Low muscle mass was defined referring to five international diagnostic criteria. The diagnostic value of Cr/CysC as a replacement indicator of muscle mass was measured using area under the curve, positive percent agreement, negative percent agreement and kappa. Cox proportional hazards regression models were developed to examine the association between Cr/CysC and risk of mortality.

**Results:**

This cohort study of 3,741 adults comprised 1,823 females (48.73%), with a weighted mean (SE) age of 44.46 (0.43) years. The positive percent agreement of Cr/CysC for the diagnosis of low muscle mass was poor (40.23–58.74%), except for Foundation of the National Institute of Health (FNIH) criteria (80.90–58.97%). But the negative percent agreement of Cr/CysC for the diagnosis of low muscle mass was high (males: 62.15–88.17%; females: 55.26–82.30%). Moreover, the risk of death was reduced by 2% per 0.01 unit increase in Cr/CysC (aHR, 0.98; 95% CI, 0.98–0.99, *P* < 0.001).

**Conclusions:**

Cr/CysC performed well not only in identifying non-sarcopenia cases, especially when based on FNIH diagnostic criteria, but also in revealing a positive association with higher risk of mortality. The optimal cut-off values for Cr/CysC were <1.0 in males and <0.8 in females. Expanding the use of Cr/CysC would allow for early and targeted treatment of sarcopenia.

## Introduction

Sarcopenia was first proposed in the 1980s as an age-related decline in lean body mass affecting mobility, nutritional status, and independence (1). Sarcopenia exerts adverse health outcomes, including falls, functional decline, frailty, impaired quality of life, and mortality (2, 3). As a result, there are a growing number of clinical trials seeking ways to improve prognosis and reduce the medical burden for patients with sarcopenia. However, the definition of low muscle mass, which is fundamental and vital for the diagnosis of sarcopenia, has not been universally agreed (4–8).

Although muscle mass can be quantified by various imaging techniques, such as dual-energy X-ray absorptiometry (DXA) and bioelectrical impedance analysis, the cut-off value of muscle mass based on the aforementioned techniques is considered method- and device-dependent, and ethnicity-dependent (9). Moreover, using these techniques routinely to assess muscle mass is not only wasteful of resources, but also impractical for patients with unstable status. Therefore, there is a requirement for a more reproducible, affordable, and widely available method for identifying patients with low muscle mass.

In 2017, Kashani et al. from the Mayo Clinic proposed the serum Creatinine/Cystatin C (Cr/CysC) as a simplified indicator to identify patients with low muscle mass (10). Since then, a growing number of small sample cohort studies have used Cr/CysC as a replacement indicator of muscle mass and have concluded that sarcopenia is associated with poor prognosis in specific populations, such as chronic kidney disease patients (11), and cancer patients (12). However, it is still controversial whether Cr/CysC can accurately indicate muscle mass (13). There are no standardized screening and diagnostic test studies that validate the ability of Cr/CysC to assess muscle mass and indicate poor prognosis.

Therefore, it is particularly important to conduct screening and diagnostic test studies to validate the ability of Cr/CysC to assess muscle mass and indicate poor prognosis. The aims of the current study were to assess the diagnostic value of Cr/CysC for low muscle mass, using DXA and the five consensus criteria as the reference criteria, and to evaluate the association of Cr/CysC with mortality.

## Methods

### Study population

The National Health and Nutrition Examination Survey (NHANES) is a nationally representative health survey designed and administered by the National Center for Health Statistics (NCHS) at the Centers for Disease Control and Prevention (CDC). The NHANES was designed to represent the civilian non-institutionalized US population using a complex multistage probability sampling methodology. The NCHS ethics review board has approved the NHANES protocol. Written informed consent was obtained from each participant.

Serum cystatin C was measured in a subset of 5,684 NHANES participants from 1999 to 2002, including all participants aged 60 years or older with available specimens and a 25% random sample of participants aged 12 to 59 years. This study included 3,741 of these participants who were aged ≥ 18 years, had available true DXA, serum creatinine, serum cystatin C, and body composition measurements (Supplementary Figure S1). We linked all participants to mortality data as of December 31, 2015, allowing for approximately 14 years of observation of mortality outcomes.

### Serum creatinine and cystatin C measurements

NHANES 1999–2000 creatinine measurements used the Jaffe kinetic alkaline picrate method, while NHANES 2001–2002 used a Roche coupled enzymatic analysis, at the Cleveland Clinic Foundation (CCF) laboratory. There were significant differences in results between these two measurements. We referred to the official NHANES analysis notes and corrected the values for 1999–2000 according to the CCF laboratory standards by multiplying by 1.013 and then adding 0.147 (Nhanes-1999–2000, Standard Biochemistry Profile & Hormones). All assays of cystatin C were conducted using the Dade Behring N Latex Cystatin C assay (14).

### Body composition measurements

Whole body DXA scans were acquired using Hologic QDR-4500A fan-beam densitometers (Hologic, Inc, Bedford, MA) in NHANES participants over the age of 8 years. DXA exclusion criteria included pregnancy, weight > 300 pounds (136 kg, weight limit of the scanner), height over 6 feet 5 inches (DXA table limitations), history of radiographic contrast material (barium) use in past 7 days, or nuclear medicine studies in the past 3 days.

Appendicular skeletal muscle mass (ASM, kg) and skeletal muscle mass index (SMI = ASM/height^2^, kg/m^2^) measured by DXA are among the currently accepted diagnostic indicators for low muscle mass. In the consensus proposed by Foundation of the National Institute of Health (FNIH), body mass index (BMI)-adjusted ASM [ASM/BMI, kg/(kg/m^2^)] is preferred as a diagnostic indicator.

### Definitions of low muscle mass

In this study, low muscle mass was defined referring to five international diagnostic criteria: European Working Group on Sarcopenia Older Persons (EWGSOP) 2 consensus (5); EWGSOP1 consensus (8); Asian Working Group for Sarcopenia (AWGS) consensus (4); International Working Group on Sarcopenia (IWGS) consensus (7); and FNIH consensus (6). These consensus defined sarcopenia as low muscle mass, low muscle strength, and/or low physical performance, and identified low muscle mass as a major feature of sarcopenia. Table 2 summarizes the operational definition for low muscle mass. Descriptive statistics were examined across sets of criteria (the operational definition for EWGSOP1 and IWGS are the same and described together below). In addition, we used two possible Cr/CysC cut-off values to define low muscle mass: (1) Cr/CysC criteria 1, defined by the maximum Youden index (refer to FNIH definition, Cr/CysC: male <1.0, female <0.9); (2) Cr/CysC criteria 2, defined by the sex-specific 20th percentile of Cr/CysC in participants aged 18–44 years (Cr/CysC: male <1.0, female <0.8) (15, 16).

### Mortality ascertainment

We used data from the NHANES public-use linked mortality file, which was linked by the NCHS to the National Death Index. Case definitions for underlying causes of death were according to the *International Classification of Diseases, Tenth Revision (ICD-10)* (17).

### Covariates assessment

Other covariates included age, sex, race/ethnicity, height, weight, BMI, total fat mass, total skeletal muscle mass and laboratory tests. According to the 1997 standards from the US Office of Management and Budget, race/ethnicity was categorized as Hispanic (including Mexican and non-Mexican Hispanic), non-Hispanic black, non-Hispanic white, and other race/ethnicity. Body weight and height of each participant were measured using standard methods and BMI was calculated as weight in kilograms divided by squared height in meters. Total fat mass and total skeletal muscle mass were measured by DXA. Blood samples were collected and tested using prescribed methods. Blood urea nitrogen (BUN), total cholesterol (TC), triglycerides (TG), high-density lipoprotein cholesterol (HDL-C), low-density lipoprotein cholesterol (LDL-C), fasting blood-glucose (FPG), hemoglobin A1c (HbA1c), serum albumin and hemoglobin (Hb), neutrophile granulocyte (NE) were included for analysis.

### Statistical analysis

We used the NHANES recommended weights to account for planned oversampling of specific groups (18). Descriptive weighted statistics were used to describe the study population, with categorical characteristics summarized as counts (percentages) and continuous characteristics summarized as means [SE (standard error)]. Continuous variables were compared by the two independent samples *t*-test, and categorical variables were compared by the chi-square test or Fisher's exact test.

Sensitivity and specificity were not determined, as they were not applicable in the absence of a gold standard defining low muscle mass. Furthermore, positive predictive value, negative predictive value, and likelihood ratios could not be calculated because the status of the participants (as determined by the reference criteria) was unknown. The diagnostic value of Cr/CysC as a replacement indicator of muscle mass was measured using four indicators of performance: (1) area under the curve (AUC) of the Receiver Operating Characteristic (ROC) curve; (2) positive percent agreement (PPA; the number of participants who were categorized as low muscle mass by both Cr/CysC and reference criteria divided by the number of participants who were categorized as low muscle mass by reference criteria, analogous to a sensitivity calculation); (3) negative percent agreement (NPA; the number of participants who were categorized into the no low muscle mass category by both Cr/CysC and reference criteria divided by the number of participants who were similarly categorized by the reference criteria, analogous to a specificity calculation; (4) Cohen's kappa (a kappa value of 1 indicates perfect agreement, whereas a kappa value of 0 indicates agreement equivalent to chance).

Logistic regression models were developed to examine the association between Cr/CysC and low muscle mass. The dependent variables were the five diagnostic criteria defining the low muscle mass status. Considering the possibility of overfitting, we quantified the multicollinearity between variables using variance inflation factors (VIF; Supplementary Table S1). Variables with VIF > 10 or a missing data proportion > 5% were excluded. Finally, the model was adjusted for BUN, TC, HbA1c, serum albumin, Hb, NE, and total fat mass. Multivariate logistic regression model results were expressed as odds ratios (OR) and 95% confidence intervals (95% CI). Furthermore, stratified analyses and interaction analyses were used to examine whether the association differed by age, sex, race/ethnicity, and obesity status.

Cox proportional hazards regression models were used to estimate hazard ratios (HR), and 95% CI for the associations between Cr/CysC and risk of mortality. In the adjusted model, we adjusted for age, sex, race/ethnicity, BUN, TC, HbA1c, serum albumin, Hb, NE and total fat mass. The dose-response relationship between Cr/CysC and the risk of mortality was evaluated by a restricted cubic spline. Kaplan-Meier survival analysis was used to estimate overall survival and log-rank test was used to compare survival differences between groups.

All data analyses were performed using R software (version 4.0.4; R Foundation for Statistical Computing, Vienna, Austria). A two-sided *P*-value < 0.05 indicated significance for all analyses.

## Results

### Participant characteristics and prevalence

This cohort study of 3,741 adults aged 18 years and older comprised 1,823 females (48.73%), with a weighted mean (SE) age of 44.46 (0.43) years; 1,042 participants (13.00%) were of Hispanic ancestry, 670 (10.02%) of non-Hispanic black ancestry, and 1930 (72.63%) of non-Hispanic white ancestry. During 47,139 person-years of observation [median follow-up, 14.17 years (range, 0.08–16.75) years], 1,247 deaths occurred. Study population characteristics are listed in Table 1 by sex. Males were taller, heavier and had more skeletal muscle mass and less fat mass than females (*P* < 0.001). Creatinine, cystatin C, BUN, TG, FPG, HbA1c, albumin, and Hb were significantly lower in the female group than in the male group (all *P*-values < 0.05).

**Table 1:** Characteristics of the study population according to sex (weighted).

**Characteristic ^a^**	**Total (*n* = 3741)**	**Male (*n* = 1918)**	**Female (*n* = 1823)**	***P*-value**
**Weighted distributions of the participants**				
**Age, mean (SE), years**	44.46 (0.43)	43.24 (0.65)	45.62 (0.42)	0.002
**Race/Ethnicity**, ***n*** **(%)**
Hispanic	1042 (13.00)	524 (14.37)	518 (11.67)	0.178
Non-hispanic black	670 (10.02)	353 (9.64)	317 (10.39)	
Non-hispanic white	1930 (72.63)	988 (71.01)	942 (74.17)	
Other	99 (4.36)	53 (4.97)	46 (3.77)	
Height, mean (SE), cm	168.97 (0.28)	175.88 (0.31)	162.34 (0.29)	<0.001
Weight, mean (SE), kg	78.14 (0.57)	84.12 (0.67)	72.41 (0.82)	<0.001
BMI, mean (SE), kg/m^2^	27.31 (0.19)	27.13 (0.16)	27.48 (0.32)	0.328
BUN, mean (SE), mg/dL	13.93 (0.15)	14.66 (0.17)	13.22 (0.19)	<0.001
TC, mean (SE), mg/dL	200.59 (1.25)	198.21 (1.52)	202.87 (1.79)	0.044
TG, mean (SE), mg/dL *	142.81 (3.83)	158.37 (8.39)	126.76 (5.33)	0.012
HDL-C, mean (SE), mg/dL	51.11 (0.58)	46.09 (0.72)	55.91 (0.72)	<0.001
LDL-C, mean (SE), mg/dL *	121.92 (1.44)	122.43 (1.96)	121.42 (1.90)	0.695
FPG, mean (SE), mg/dL *	101.45 (1.22)	104.85 (1.54)	97.93 (1.27)	<0.001
HbA1c, mean (SE), %	5.40 (0.02)	5.44 (0.28)	5.36 (0.03)	0.027
Albumin, mean (SE), g/L	4.40 (0.01)	4.50 (0.01)	4.31 (0.02)	<0.001
Hb, mean (SE), g/L	14.47 (0.07)	15.41 (0.09)	13.57 (0.06)	<0.001
NE, mean (SE), ×103/uL	4.22 (0.06)	4.20 (0.08)	4.25 (0.07)	0.554
Total fat mass, mean (SE), kg	26.75 (0.35)	23.80 (0.35)	29.57 (0.55)	<0.001
Total SMM, mean (SE), kg	49.73 (0.32)	58.40 (0.37)	41.42 (0.31)	<0.001
ASM, mean (SE), kg	21.85 (0.16)	26.27 (0.19)	17.61 (0.15)	<0.001
ASM/ht^2^, mean (SE), kg/m^2^	7.54 (0.04)	8.46 (0.05)	6.67 (0.06)	<0.001
ASM/BMI, mean (SE), kg/(kg/m^2^)	0.81 (0.01)	0.98 (0.00)	0.65 (0.00)	<0.001
Cr, mean (SE), mg/dL	0.90 (0.01)	1.01 (0.01)	0.79 (0.01)	<0.001
CysC, mean (SE), mg/dL	0.88 (0.01)	0.89 (0.01)	0.86 (0.01)	0.008
Cr/CysC, mean (SE)	1.04 (0.01)	1.15 (0.01)	0.94 (0.01)	<0.001

*BMI, body mass index; BUN, blood urea nitrogen; TC, total cholesterol; TG, triglyceride; HDL-C, high-density lipoprotein cholesterol; LDL-C, low-density lipoprotein cholesterol; FPG, fasting blood-glucose; HbA1c, hemoglobin A1c; Hb, hemoglobin; NE, Neutrophile granulocyte; SMM, skeletal muscle mass; ASM, appendicular skeletal muscle mass; ht, Height; Cr, creatinine; CysC, Cystatin C*.

*^**a**^All means and SEs for continuous variables and percentages for categorical variables were weighted, with the exception of the number of participants. Because all numbers were rounded, percentages may not total 100%*.

*Data missing > 5%.

The various operational definitions and prevalence (weighted) of low muscle mass are presented in Table 2. The prevalence of FNIH-defined low muscle mass (8.41%) is smaller than that defined by the EWGSOP2 (11.71%), EWGSOP1/IWGS (17.36%), and AWGS (10.46%) in the total population. The prevalence of low muscle mass defined by Cr/CysC criteria 2 (male <1.0, female <0.8) was more consistent with the international consensus than the prevalence defined by Cr/CysC criteria 1 (male <1.0, female <0.9).

**Table 2:** Summary of operational definition and prevalence (weighted) for low muscle mass by sex.

**Diagnosis criteria**		**Operational definition**		**Prevalence**, ***n*** **(%)**		
		**ASM** **(kg)**	**SMI** **(kg/m^2^) ^a^**	**Male** **(*n* = 1918)**	**Female** **(*n* = 1823)**	**Total** **(*n* = 3741)**
EWGSOP2 (2019)	Male	<20	<7.00	268 (8.88)	312 (14.42)	580 (11.71)
	Female	<15	<5.50			
EWGSOP1 (2010)/IWGS (2011)	Male	-	<7.23	382 (14.36)	407 (20.23)	789 (17.36)
	Female	-	<5.67			
AWGS (2019)	Male	-	<7.00	268 (8.88)	265 (11.97)	533 (10.46)
	Female	-	<5.40			
FNIH (2014)	Male	<19.75	<0.789	386 (9.43)	302 (7.43)	688 (8.41)
	Female	<15.02	<0.512			
Cr/CysC criteria 1 ^b^	Male	Cr/CysC <1.0		622 (23.21)	1043 (45.97)	1665 (34.83)
	Female	Cr/CysC <0.9				
Cr/CysC criteria 2 ^C^	Male	Cr/CysC <1.0		622 (23.21)	591 (21.99)	1213 (22.59)
	Female	Cr/CysC <0.8				

*ASM, appendicular skeletal muscle mass; SMI, skeletal muscle mass index; EWGSOP, European Working Group on Sarcopenia Older Persons; IWGS, International Working Group on Sarcopenia; AWGS, Asian Working Group for Sarcopenia; FNIH, Foundation of the National Institute of Health; Cr, creatinine; CysC, Cystatin C*.

*^**a**^SMI, ASM/height^2^ or ASM/body mass index (FNIH)*.

*^**b**^The cut-off values were defined by the maximum Youden index (refer to FNIH definition, Cr/CysC: Male <1.0, Female <0.9)*.

*^**C**^The cut-off values were defined by the sex-specific 20th percentile of Cr/CysC in participants aged 18–44 years (Cr/CysC: Male <1.0, Female <0.8)*.

### Diagnostic value of Creatinine/Cystatin C

The statistical analysis (weighted) of positive percent agreement, negative percent agreement, AUC, cut-off value, kappa value between Cr/CysC or Cr/CysC criteria and other five diagnostic criteria is summarized in Table 3.

**Table 3:** Agreement comparison of Cr/CysC criteria with other diagnosis criteria for low muscle mass (weighted).

**Diagnosis criteria**		**PPA (%)**	**NPA (%)**	**AUC**	**cut-off ^a^**	**kappa**
EWGSOP2 (2019)	Male	42.38	88.17	0.70	1.19	0.08
	Female	40.82	80.32	0.63	0.98	0.09
EWGSOP1 (2010)/IWGS (2011)	Male	43.57	83.61	0.66	1.19	0.12
	Female	58.74	55.26	0.58	0.90	0.10
AWGS (2019)	Male	42.38	88.17	0.70	1.19	0.08
	Female	40.23	80.28	0.63	0.98	0.07
FNIH (2014)	Male	80.90	62.15	0.77	1.00	0.26
	Female	58.97	82.30	0.74	0.90	0.13

*EWGSOP, European Working Group on Sarcopenia Older Persons; IWGS, International Working Group on Sarcopenia; AWGS, Asian Working Group for Sarcopenia; FNIH, Foundation of the National Institute of Health; Cr, creatinine; CysC, Cystatin C; PPA, positive percent agreement: the number of participants who were categorized as low muscle mass by both Cr/CysC and reference criteria divided by the number of participants who were categorized as low muscle mass by reference criteria; NPA, negative percent agreement: the number of participants who were categorized into the no low muscle mass category by both Cr/CysC and reference criteria divided by the number of participants who were similarly categorized by the reference criteria; AUC, area under the curve*.

*^**a**^the cut-off values of Cr/CysC Criteria were defined by the maximum Youden index (refer to consensus definition)*.

The positive percent agreement of Cr/CysC for the diagnosis of low muscle mass was poor (male: 42.28%−43.57%; female: 40.23%−58.74%) for the rest of the diagnostic criteria, except for FNIH criteria (male: 80.90%; female: 58.97%). But the negative percent agreement of Cr/CysC for the diagnosis of low muscle mass was high (male: 62.15%−88.17%; female: 55.26%−82.30%).

Further analysis of the AUC revealed that Cr/CysC had a better diagnostic value for low muscle mass when using the diagnostic criteria of EWGSOP2 (AUC = 0.70, 0.63), EWGSOP1/IWGS (AUC = 0.66, 0.58), AWGS (AUC = 0.70, 0.63) and FNIH (AUC = 0.77, 0.74). The highest AUC was available for Cr/CysC when using the diagnostic criteria of FNIH, at which point the cut-off value of Cr/CysC was 1.0 for males and 0.9 for females (Cr/CysC criteria 1).

In addition, the kappa values between Cr/CysC and other diagnostic criteria were moderate, with a range of 0.08–0.26 in males and 0.07–0.13 in females (Table 3), which were higher than the kappa values between FNIH and the other diagnostic criteria (male: 0.12–0.13; female: 0.01–0.03, Supplementary Table S2). Furthermore, the highest kappa values between both Cr/CysC criteria and FNIH (male: 0.26; female: 0.12–0.16, Supplementary Table S2).

### The association between Creatinine/Cystatin C and low muscle mass

**Figure 1:**
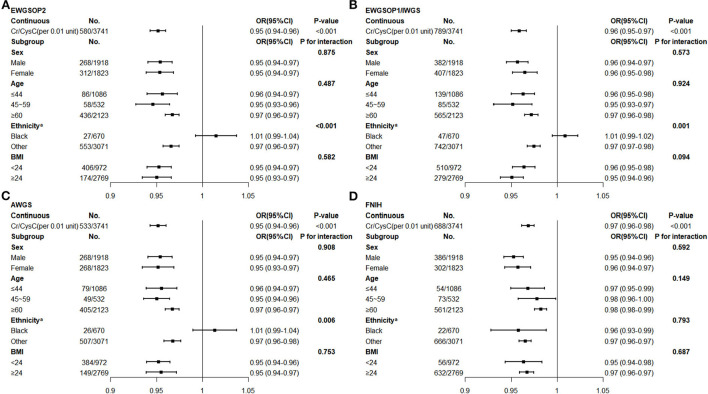
Multivariate Logistic Regression of Creatinine/Cystatin C and Low Muscle Mass (Weighted)^***b***^ **(A)** Low muscle mass defined by the EWGSOP 2 expert consensus. **(B)** Low muscle mass defined by the EWGSOP 1/IWGS expert consensus. **(C)** Low muscle mass defined by the AWGS expert consensus. **(D)** Low muscle mass defined by the FNIH expert consensus. EWGSOP, European Working Group on Sarcopenia Older Persons; IWGS, International Working Group on Sarcopenia; AWGS, Asian Working Group for Sarcopenia; FNIH, Foundation of the National Institute of Health; Cr, creatinine; CysC, Cystatin C; BMI, body mass index. ^**a**^Adjusted blood urea nitrogen, total cholesterol, neutrophile granulocyte. ^**b**^Adjusted total fat mass, blood urea nitrogen, total cholesterol, hemoglobin A1c, albumin, hemoglobin, neutrophile granulocyte.

Through multivariate logistic regression analysis (weighted), we observed that Cr/CysC was significantly associated with low muscle mass defined by different diagnostic criteria (Figure 1). The prevalence of low muscle mass decreased significantly per 0.01 increase in Cr/CysC [EWGSOP2 (aOR, 0.95; 95% CI, 0.94–0.96, *P* < 0.001), EWGSOP1/IWGS (aOR, 0.96; 95% CI, 0.95–0.97, *P* < 0.001), AWGS (aOR, 0.95; 95% CI, 0.94–0.96, *P* < 0.001), FNIH (aOR, 0.97; 95% CI, 0.96–0.98, *P* < 0.001)]. Further stratified and interaction analyses were conducted for age, sex, race/ethnicity, and obesity status. We found that when referring to the FNIH criteria, such results remained significant in the non-Hispanic black participants (aOR, 0.97; 95% CI, 0.96–0.97, *P* < 0.001, Figure 1D), but when other consensus was used as the diagnostic criterion, the association between increased Cr/CysC and the prevalence of low muscle mass was not significant in the non-Hispanic black participants (all *P* > 0.05, Figures 1A-C). The association between Cr/CysC and low muscle mass did not differ by age, sex, or obesity status (all *P* for interactions > 0.05).

### Associations between Creatinine/Cystatin C and mortality

**Figure 2:**
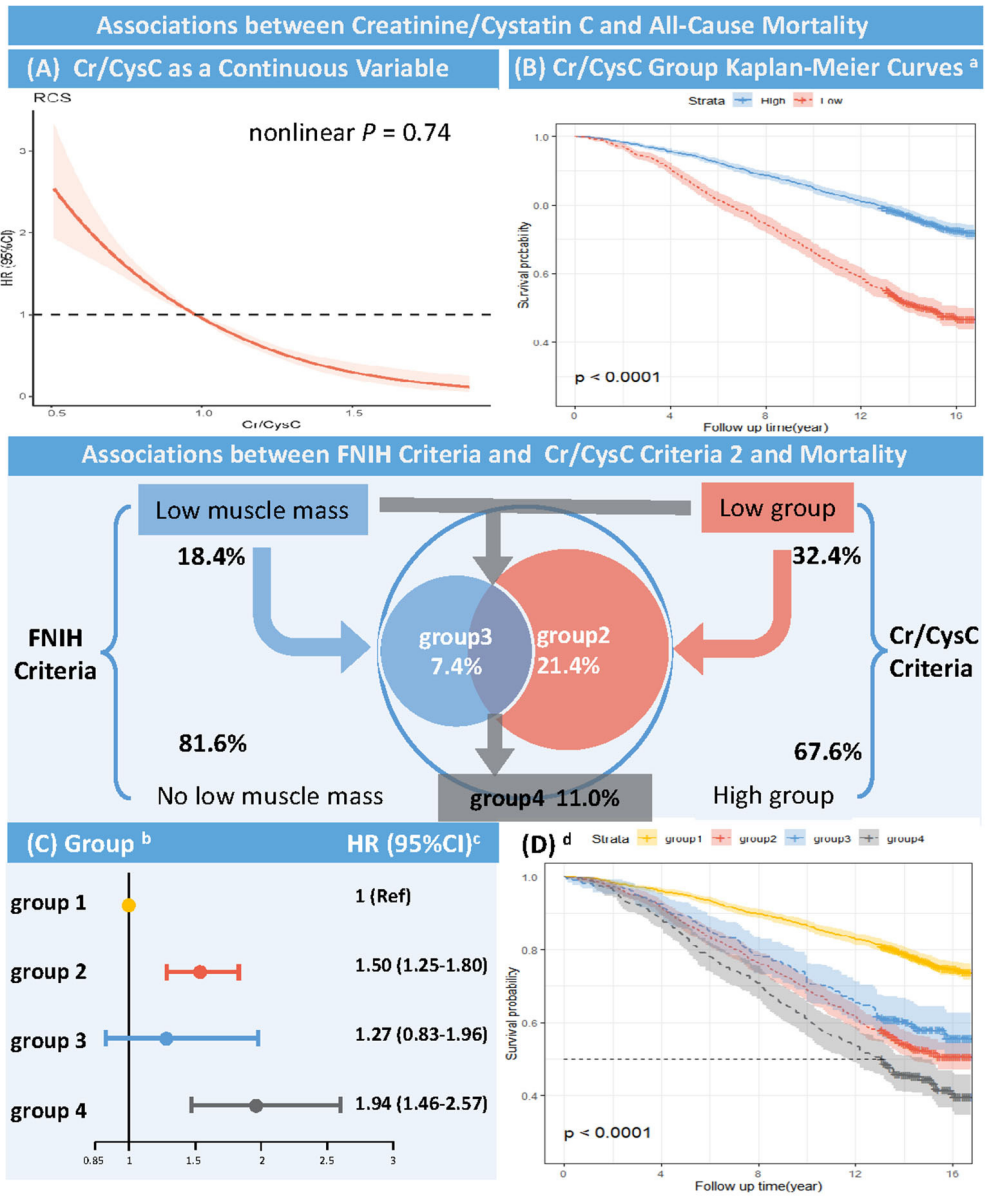
Association between Creatinine/Cystatin C and All-cause Mortality (Unweighted) RCS, restricted cubic spline; Cr, creatinine; CysC, Cystatin C; FNIH, Foundation of the National Institute of Health. **(A)** RCS of HR and 95% CI for the association between Cr/CysC (continuous) and all-cause mortality (Nonlinear P = 0.74). **(B)** Kaplan-Meier curves according to Cr/CysC criteria (unweighted). ^**a**^The cut-off values were defined by the sex-specific 20th percentile of Cr/CysC in participants aged 18–44 years (Cr/CysC: Male <1.0, Female <0.8). High group: Cr/CysC criteria 2 define no low muscle mass; Low group: Cr/CysC criteria 2 define low muscle mass (Log-rank test, P < 0.001). **(C)** Multivariate Cox proportional risk model (weighted). ^**b**^Group 1 = both Cr/CysC and FNIH criteria define no low muscle mass; Group 2 = only Cr/CysC criteria define low muscle mass; Group 3 = only FNIH criteria define low muscle mass; Group 4 = both Cr/CysC and FNIH criteria define low muscle mass. The cut-off values were defined by the sex-specific 20th percentile of Cr/CysC in participants aged 18–44 years (Cr/CysC: Male <1.0, Female <0.8). ^**c**^Adjusted age, sex, race/ethnicity, total fat mass, blood urea nitrogen, total cholesterol, hemoglobin A1c, albumin, and hemoglobin. **(D)** Kaplan-Meier curves according to Cr/CysC and FNIH criteria. d Group 1 = both Cr/CysC and FNIH criteria define no low muscle mass; Group 2 = only Cr/CysC criteria define low muscle mass; Group 3 = only FNIH criteria define low muscle mass; Group 4 = both Cr/CysC and FNIH criteria define low muscle mass. The cut-off values were defined by the sex-specific 20th percentile of Cr/CysC in participants aged 18–44 years (Cr/CysC: Male <1.0, Female <0.8) (Log-rank test, P < 0.001).

During follow-up, the risk of participants' all-cause death decreased with increasing Cr/CysC in a linear relationship (non-linear *P* = 0.74, Figure 2). The risk of all-cause mortality was reduced by 2% per 0.01 unit increase in Cr/CysC (aHR, 0.98; 95% CI, 0.98–0.99, *P* < 0.001, Supplementary Table S3).

Kaplan-Meier curves showed higher all-cause mortality in the low Cr/CysC group (male <1.0, female <0.8) than in the high Cr/CysC group during follow-up (log-rank test, *P* < 0.001, Figure 2), with a significantly higher risk of death **(**aHR, 1.54; 95% CI, 1.30–1.84, *P* < 0.001, Supplementary Table S3).

Multivariate Cox proportional risk analysis (weighted) showed that participants with low muscle mass defined by Cr/CysC criteria 2 only (Group 2 vs. Group 1: aHR, 1.50; 95% CI, 1.25–1.80, *P* < 0.001) had a higher risk of death than those with low muscle mass defined by FNIH criteria only (Group 3 vs. Group 1: aHR, 1.27; 95% CI, 0.83–1.96, *P* = 0.275). Participants with low muscle mass defined by both Cr/CysC criteria 2 and FNIH criteria had the highest risk of death (Group 4 vs. Group 1: aHR, 1.94; 95% CI, 1.46–2.57, *P* < 0.001, Figure 2, Supplementary Table S3). Similarly, Kaplan-Meier curves showed higher all-cause mortality during follow-up in participants with low muscle mass defined by Cr/CysC criteria only than in participants with low muscle mass defined by FNIH only (log-rank test, *P* < 0.001, Figure 2). Sensitivity analysis found similar results in Cr/CysC criteria 1 (Supplementary Figure S2).

## Discussion

This study aimed to detect the clinical value of Cr/CysC in diagnosing low muscle mass in a nationally representative cohort. We found that Cr/CysC is a specific replacement indicator of low muscle mass. The optimal cut-off values for Cr/CysC were <1.0 in males and <0.8 in females. Furthermore, low muscle mass diagnosis based on this cut-off value was significantly associated with higher risks of mortality.

In our analysis, Cr/CysC performed best in the FNIH criteria (AUC: 0.77 in males; 0.74 in females) in accordance with the American population we used. The minimum AUC value observed by reference to all consensus was 0.58 for females in EWGSOP1(2010)/IWGS(2011) criteria. However, in the updated EWGSOP2(2019) criteria, the AUC value reached to 0.63 in consistence with the renewed guideline. We found Cr/CysC only had acceptable discriminatory powers in European and Asian criteria which is reasonable considering that the heterogeneity of race/ethnicity etc. in different regions worldwide. Cr/CysC criteria showed high positive percent agreement and negative percent agreement in the FNIH criteria but low positive percent agreement and high negative percent agreement in the other four diagnostic criteria. Thus, we believe that the Cr/CysC performed well in identifying participants who did not suffer from low muscle mass and who may not benefit from further assessment of muscle mass, avoiding unnecessary or disadvantageous tests and therapy. However, due to the low positive percent agreement, Cr/CysC did not have sufficient performance to prove an efficient diagnostic indicator in the general population when using EWGSOP, AWGS, or IWGS as diagnostic criteria. Furthermore, we believe that Cr/CysC was not only beneficial in excluding patients with no low muscle mass diagnosed by FNIH criteria in the population, but also had important clinical implications for the rapid identification of patients at risk of low muscle mass and for early implementation of prevention strategies.

Participants considered to have low muscle mass according to the Cr/CysC criteria and consensus criteria had little overlap, as suggested by the poor kappa values. This result is consistent with a previous study comparing FNIH criteria and other consensus criteria (19). The FNIH believes that the lack of agreement between FNIH criteria and other consensus criteria is due to the following: (1) differences in the reference population; and (2) that the FNIH criteria used ASM/BMI, whereas both the EWGSOP and IWGS criteria used ASM/height^2^. The Cr/CysC criteria in this study had the highest kappa values with the FNIH criteria, and in addition to the similarity of the reference populations, we believe that it is also because Cr/CysC was more representative of the ASM corrected for BMI, rather than height^2^. This was also demonstrated by Pearson correlation analysis (Supplementary Table S4). As previously demonstrated, low muscle mass defined according to FNIH criteria (ASM/BMI) was more strongly associated with adverse clinical outcomes than ASM/height^2^ (20). The FNIH criteria are more able to identify patients with sarcopenic obesity from among obese participants (19). In 2019, the AWGS consensus stated that the FNIH criterion may be the appropriate operational definition (4). The above evidence suggested that ASM/BMI appeared to be more accurate in the diagnosis of low muscle mass. Because the Cr/CysC performed well when FNIH criteria were used as the gold standard, it is reasonable to speculate that Cr/CysC had the same advantages as ASM/BMI.

The prevalence of low muscle mass defined by Cr/CysC criteria 2 is more in agreement with the consensus criteria than the prevalence of low muscle mass defined by Cr/CysC criteria 1. Also, the kappa value of Cr/CysC criteria 2 in relation to the consensus criteria was higher compared to Cr/CysC criteria 1. Therefore, we believe that using the sex-specific 20th percentile of Cr/CysC in participants aged 18–44 years defined as low muscle mass (male <1.0, female <0.8) seemed to be a more appropriate criterion for Cr/CysC.

Our subgroup analysis showed that Cr/CysC was not associated with low muscle mass in the non-Hispanic black participants, using the EWGSOP, AWGS, and IWGS criteria. However, when low muscle mass was diagnosed by FNIH, for every 0.01 unit increase in Cr/CysC was associated with a 3% decrease in the prevalence of low muscle mass in non-Hispanic blacks. This may be related to the different diagnostic indicator and reference populations. Due to the presence of African Americans, the FNIH considers the influence of the black population when defining low muscle mass, whereas the reference populations of other consensus have a lower percentage of blacks. Therefore, with reference to the results of the FNIH consensus, we believe that Cr/CysC criteria had the same ability to diagnose low muscle mass in the non-Hispanic black population. Considering that the muscle mass of blacks is fundamentally different from that of other races, this racial difference cannot be ignored (9). In addition, our results showed that the efficacy of Cr/CysC in diagnosing low muscle mass was not affected by age, sex, and even BMI. In recent years, sarcopenia obesity has been increasingly gaining attention and is considered to have a worse prognosis compared to sarcopenia (21). If the efficacy of Cr/CysC in diagnosing low muscle mass is equal in obese and non-obese populations, the underestimation of sarcopenia in the obese population can be corrected.

The relationship between patients with low muscle mass diagnosed by Cr/CysC criteria and FNIH criteria and all-cause mortality was further analyzed. We found that patients with low muscle mass diagnosed by Cr/CysC criteria alone had a higher risk of mortality than patients with low muscle mass diagnosed by FNIH criteria alone. This finding suggested that the risk of mortality from low muscle mass diagnosed by Cr/CysC criteria was higher in a community-based US population. Therefore, treatment of low muscle mass based on Cr/CysC criteria may have greater benefit. This may be due to the high negative percent agreement of the Cr/CysC criteria, which makes it possible to distinguish more accurately between healthy people and potentially sick people. However, it cannot accurately distinguish between truly healthy people and those with truly low muscle mass. Therefore, treatment and intervention based on Cr/CysC criteria may increase the health economic burden. In addition, other factors contributing to the decline in Cr/CysC cannot be ruled out as contributing to the poorer prognosis of patients. Still, no simple lab index to date can have both diagnostic and prognostic values for sarcopenia, our Cr/CysC criteria will provide insights into the clinical practice of sarcopenia.

The primary limitation of this cohort study was that it used four different regional consensus to assess the effectiveness of Cr/CysC based on US populations. Although the Cr/CysC is a valid and reliable replacement indicator for muscle mass according to the FNIH, it is necessary to further assess the validity of Cr/CysC using a population in a region corresponding to the relevant consensus. Furthermore, among the various techniques for measuring muscle mass, we only chose DXA as the standard measure. However, DXA is also the most respected measurement tool among these five consensus in recent years. Finally, in addition to the factors we included in the analysis, there are many other indicators that may affect Cr and CysC that were not considered, such as protein intake, hormone levels. However, due to the limitation of the database, we were not able to obtain the above indicators accurately. We will pay more attention to the collection and analysis of relevant indicators in future studies.

## Conclusion

Creatinine/Cystatin C performed well not only in identifying non-sarcopenia cases, especially when based on FNIH diagnostic criteria, but also in revealing a positive association with higher risk of all-cause mortality. The optimal cut-off values for Cr/CysC were <1.0 in males and <0.8 in females. Expanding the use of Creatinine/Cystatin C would allow for early and targeted treatment of sarcopenia. Provide a theoretical basis for a more standardized and consistent diagnosis of sarcopenia.

## Data availability statement

The datasets presented in this study can be found in online repositories. The names of the repository/repositories and accession number(s) can be found below: the National Health and Nutrition Examination Survey repository [https://www.cdc.gov/nchs/nhanes/].

## Ethics statement

The studies involving human participants were reviewed and approved by the National Center for Health Statistics ethics review board. The patients/participants provided their written informed consent to participate in this study.

## Author contributions

KH had full access to all the data in the study and takes responsibility for the integrity of the data and the accuracy of the data analysis. Study concept, design, and drafting of the manuscript: SS, YJ, and WC. Acquisition, analysis, or interpretation of data and critical revision of the manuscript for important intellectual content: all authors. Statistical analysis: KH and SS. Obtained funding: KC. Administrative, technical, or material support: YL. Study supervision: YL and KC. All authors contributed to the article and approved the submitted version.

## Funding

This work was supported by the Fujian Provincial Natural Science Foundation (2022J011503) and Longyan City Science and Technology Plan Project (2021LYF17025). The funder had no role in the design and conduct of the study, collection, management, analysis, interpretation of the data, preparation, review, approval of the manuscript, and decision to submit the manuscript for publication.

## Conflict of interest

The authors declare that the research was conducted in the absence of any commercial or financial relationships that could be construed as a potential conflict of interest.

## Publisher's note

All claims expressed in this article are solely those of the authors and do not necessarily represent those of their affiliated organizations, or those of the publisher, the editors and the reviewers. Any product that may be evaluated in this article, or claim that may be made by its manufacturer, is not guaranteed or endorsed by the publisher.
