# Supplementary material for: Diagnostic and prognostic value of the Creatinine/Cystatin C ratio for low muscle mass evaluation among US adults
Source: Front Nutr. 2022 Aug 9;9:897774. doi: 10.3389/fnut.2022.897774 (PMC9398338; doi:10.3389/fnut.2022.897774)
Supplement: Supplementary file 1 [file Data_Sheet_1.pdf]

Supplemental Material

Supplementary Table S1 Multicollinearity Analysis

Variables	VIF [a] (ASM)	VIF (SMI)
Cr/CysC	3.83	6.88
Sex	4.46	3.68
Age	12.75	8.17
Race/Ethnicity	29.27	24.93
Total fat mass	2.46	1.95
BUN	3.97	6.19
TC	25484.41	53609.74
TG	5338.53	5168.14
HDL-C	3358.36	3830.20
LDL-C	21839.49	50662.91
FPG	9.85	7.50
HbA1c	9.18	7.19
Albumin	4.76	6.98
Hb	5.89	3.80
NE	2.28	3.51

Abbreviations: VIF, variance inflation factor; ASM, appendicular skeletal muscle mass; SMI, skeletal muscle mass index; Cr, creatinine; CysC, Cystatin C; BUN, blood urea nitrogen; TC, total cholesterol; TG, triglyceride; HDL-C, high-density lipoprotein cholesterol; LDL-C, low-density lipoprotein cholesterol; FPG, fasting blood-glucose; HbA1c, hemoglobin A1c; Hb, hemoglobin; NE, Neutrophile granulocyte.

[a] The VIF method was used to examine multicollinearity, and VIF > 10 suggested multicollinearity in model.

Supplementary Table S2 Kappa Values between Different Operational Definition (Weighted)

Diagnosis Criteria		kappa (Cr/CysC) [a]	kappa (Cr/CysC criteria 1) [b]	kappa (Cr/CysC criteria 2) [c]	kappa (FNIH) [d]
**EWGSOP2 (2019)**	Male	0.085	0.136	0.136	0.122
	Female	0.089	0.102	0.083	0.028
	Total	0.129	0.129	0.107	0.067
**EWGSOP1 (2010)/IWGS (2011)**	Male	0.117	0.129	0.129	0.128
	Female	0.097	0.085	0.050	0.008
	Total	0.097	0.117	0.087	0.060
**AWGS (2019)**	Male	0.085	0.136	0.136	0.122
	Female	0.073	0.081	0.090	0.026
	Total	0.108	0.109	0.112	0.070
**FNIH (2014)** [e]	Male	0.261	0.260	0.260	-
	Female	0.126	0.118	0.157	-
	Total	0.114	0.165	0.209	-

Abbreviations: EWGSOP, European Working Group on Sarcopenia Older Persons; IWGS, International Working Group on Sarcopenia; AWGS, Asian Working Group for Sarcopenia; FNIH, Foundation of the National Institute of Health; Cr, creatinine; CysC, Cystatin C.

[a] the cut-off values were defined by the maximum Youden index (refer to consensus definition).

[b] the cut-off values were defined by the maximum Youden index (refer to FNIH definition, Cr/CysC: Male < 1.0, Female < 0.9).

[C] the cut-off values were defined by the sex-specific 20th percentile of Cr/CysC in participants aged 18-44 years (Cr/CysC: Male < 1.0, Female < 0.8).

[d] the cut-off values were defined by FNIH (SMI: Male < 0.789, Female < 0.512).

[e] the cut-off values for Cr/CysC criteria 1 were defined by reference to FNIH criteria, but since only two decimal places were retained, the population was divided differently from that defined directly by the maximum Youden index.

Supplementary Table S3 Multivariate Cox Proportional Risk Model (Weighted)

	Cr/CysC criteria 1 [b]			Cr/CysC criteria 2 [c]		
**Models [a]**	**Event**	**HR (95% CI)**	***P*-value**	**Event**	**HR (95% CI)**	***P*-value**
**Continuous**						
Cr/CysC (per 0.01 unit)	1247/3741	0.98 (0.98-0.99)	<0.001	1247/3741	0.98 (0.98-0.99)	<0.001
**Cr/CysC group**						
High	487/2076	1 (Ref)		636/2528	1 (Ref)	
Low	760/1665	1.53 (1.25-1.87)	<0.001	611/1213	1.54 (1.30-1.84)	<0.001
**Cr/CysC-FNIH [d]**						
Group 1	396/1872	1 (Ref)		520/2251	1 (Ref)	
Group 2	502/1181	1.49 (1.18-1.89)	0.001	378/802	1.50 (1.25-1.80)	<0.001
Group 3	91/204	1.30 (0.89-1.90)	0.178	116/277	1.27 (0.83-1.96)	0.275
Group 4	258/484	1.93 (1.41-2.64)	<0.001	233/411	1.94 (1.46-2.57)	<0.001

Abbreviations: Cr, creatinine; CysC, Cystatin C; FNIH, Foundation of the National Institute of Health.

[a] adjusted age, sex, race/ethnicity, total fat mass, blood urea nitrogen, total cholesterol, hemoglobin A1c, albumin, hemoglobin, neutrophile granulocyte.

[b] the cut-off values were defined by the maximum Youden index (refer to FNIH definition, Cr/CysC: Male < 1.0, Female < 0.9).

[c] the cut-off values were defined by the sex-specific 20th percentile of Cr/CysC in participants aged 18-44 years (Cr/CysC: Male < 1.0, Female < 0.8).

[d] Group 1 = both Cr/CysC and FNIH criteria define no low muscle mass; Group 2 = only Cr/CysC criteria define low muscle mass; Group 3 = only FNIH criteria define low muscle mass; Group 4 = both Cr/CysC and FNIH criteria define low muscle mass.

Supplementary Figure S1 Flowchart of Participants Selection for Analytic

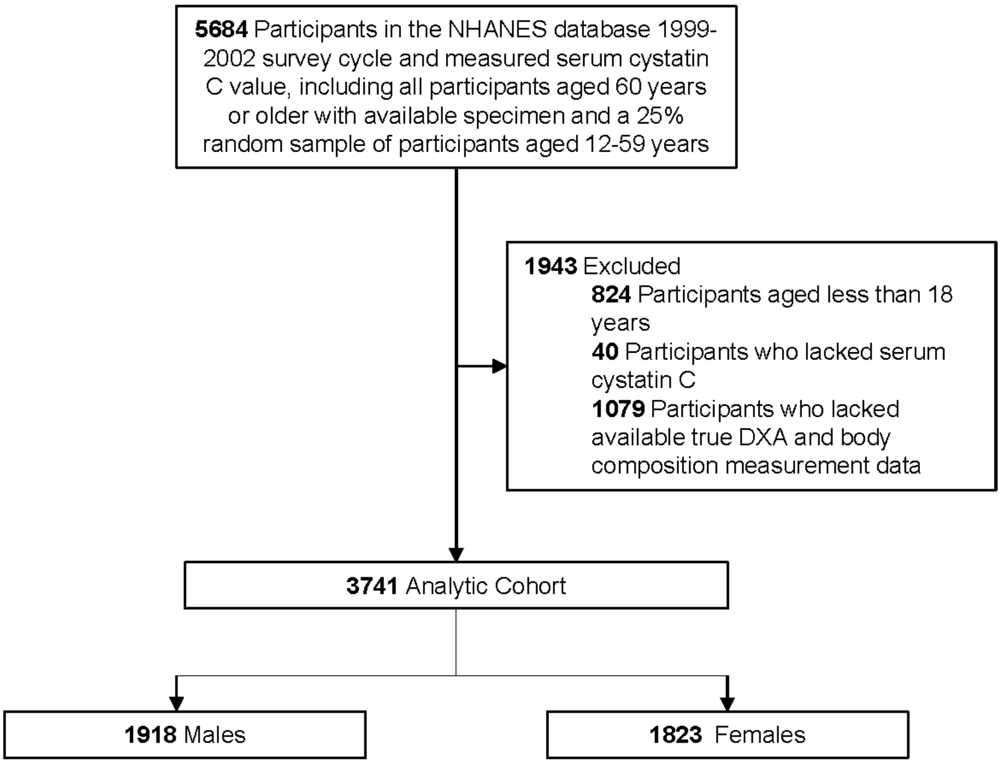

No.	Cr/CysC criteria (cut-off=1.0)	
FNIH[a]	Low group	High group
Low muscle mass	230	156
No low muscle mass	392	1140

No.	Cr/CysC criteria (cut-off=0.8)	
FNIH[a]	Low group	High group
Low muscle mass	181	121
No low muscle mass	410	1111

[a] Take FNIH as an example.

Supplementary Figure S2 Association between Cr/CysC Criteria 1 and All-Cause Mortality

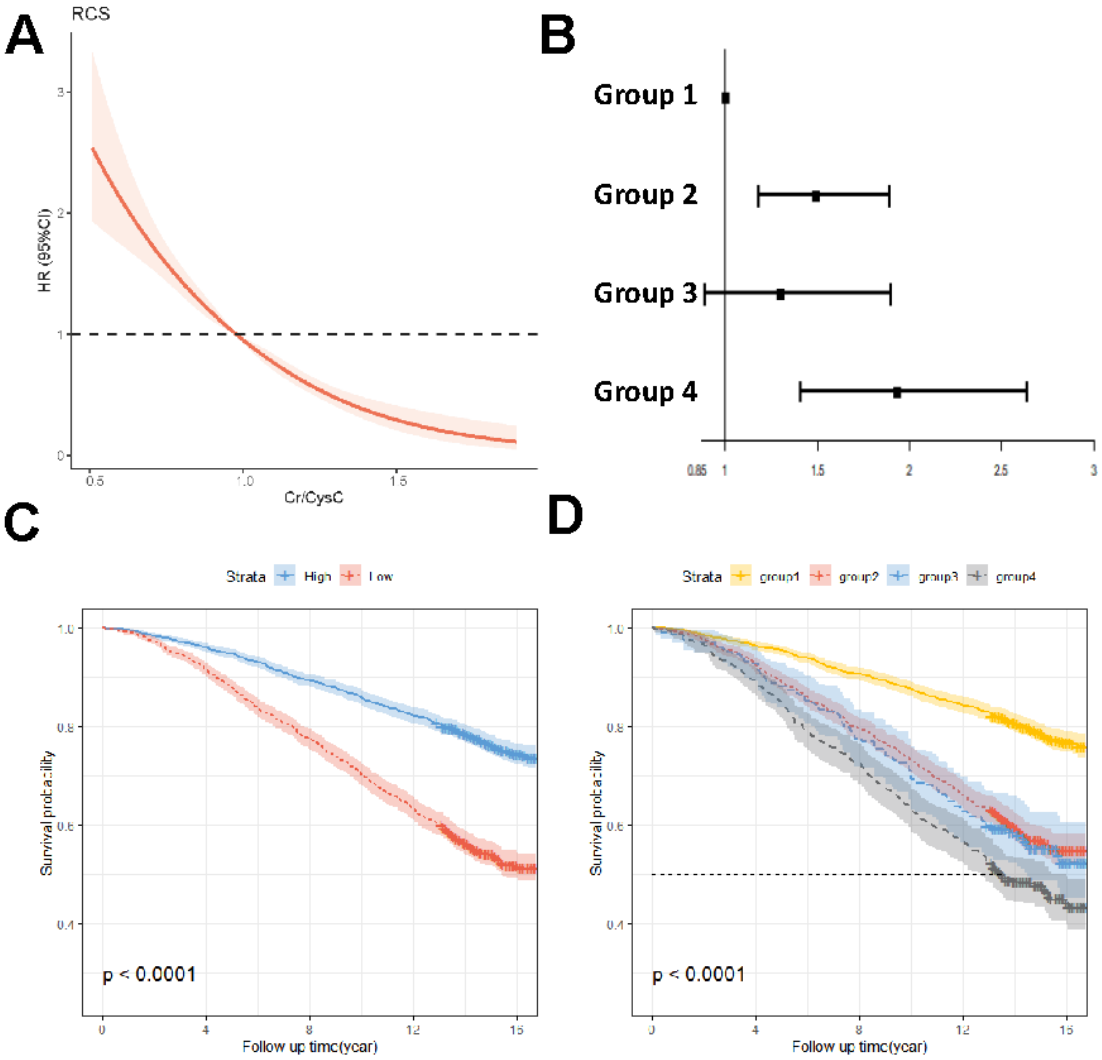

Abbreviations: RCS, restricted cubic spline; Cr, creatinine; CysC, Cystatin C; LMM, low muscle mass.

(A) RCS of HR and 95% CI for the association between Cr/CysC (continuous) and all-cause mortality (unweighted) (Nonlinear $P = 0.74$).

(B) Multivariate Cox proportional risk model (weighted). Group 1 = both Cr/CysC and FNIH criteria define no low muscle mass; Group 2 = only Cr/CysC criteria define low muscle mass; Group 3 = only FNIH criteria define low muscle mass; Group 4 = both Cr/CysC and FNIH criteria define low muscle mass. The cut-off values were defined by the maximum Youden index (refer to FNIH definition, Cr/CysC: Male < 1.0, Female < 0.9). Adjusted age, sex, race/ethnicity, total fat mass, blood urea nitrogen, total cholesterol, hemoglobin A1c, albumin, hemoglobin.

(C) Kaplan-Meier curves according to Cr/CysC criteria (unweighted). LMM = 0: Cr/CysC criteria define no low muscle mass; LMM = 1: Cr/CysC criteria define low muscle mass. The cut-off values were defined by the maximum Youden index (refer to FNIH definition, Cr/CysC: Male < 1.0, Female < 0.9) (Log-rank test, $P < 0.001$).

(D) Kaplan-Meier curves according to Cr/CysC and FNIH criteria (unweighted). Group 1 = both Cr/CysC and FNIH criteria define no low muscle mass; Group 2 = only Cr/CysC criteria define low muscle mass; Group 3 = only FNIH criteria define low muscle mass; Group 4 = both Cr/CysC and FNIH criteria define low muscle mass. The cut-off values were defined by the maximum Youden index (refer to FNIH definition, SI: Male < 1.0, Female < 0.9) (Log-rank test, $P < 0.001$).

Supplementary Table S4 Pearson correlation analysis

	**Cr/CysC**	**ASM, kg**	**ASM/ht^2, kg/m^2**	**ASM/BMI, kg/(kg/m^2)**
Cr/CysC	1	0.46*	0.38*	0.59*
ASM, kg	0.46*	1	0.92*	0.72*
ASM/ht^2, kg/m^2	0.38*	0.92*	1	0.49*
ASM/BMI, kg/(kg/m^2)	0.59*	0.72*	0.49*	1

Abbreviations: BMI, body mass index; ASM, appendicular skeletal muscle mass; ht, Height; Cr, creatinine; CysC, Cystatin C.

* $P < 0.001$
